# Hybrid Hollow Fiber Nanofiltration–Calcite Contactor: A Novel Point-of-Entry Treatment for Removal of Dissolved Mn, Fe, NOM and Hardness from Domestic Groundwater Supplies

**DOI:** 10.3390/membranes9070090

**Published:** 2019-07-19

**Authors:** Maryam Haddad, Benoit Barbeau

**Affiliations:** NSERC-Industrial Chair on Drinking Water, Polytechnique de Montréal, Montréal, QC H3T 1J4, Canada

**Keywords:** hollow fiber nanofiltration, NF270 and NF90 membranes, hard groundwater, manganese, iron and natural organic matter removal, calcite contactor

## Abstract

Groundwater (GW) is one of the main potable water sources worldwide. However, the presence of undesirable compounds and particularly manganese (Mn) and iron (Fe) (mainly co-existing in GWs) are considered as objectionable components of potable water for both health and aesthetic issues. As such, individual dwellings supplied by domestic wells are especially threatened by these issues. Current domestic treatment technologies are complicated to operate and even dangerous if improperly maintained (e.g., catalytic filtration) or consume salts and produce spent brine which pollutes the environment (i.e., ion exchange resins). Therefore, it is of prime importance to design a simple and compact, yet robust, system for Mn and Fe control of the domestic GW sources, which can reliably guarantee the desired Mn limit in the finished water (20μg/L). In the course of this study, we demonstrated, for the first time, that a hybrid hollow fiber nanofiltration (HFNF)–calcite contactor process is a promising alternative for treating domestic GWs with elevated levels of Mn, Fe, natural organic matter (NOM) and hardness. The efficacy of the HFNF membranes in terms of removal of Mn, Fe, NOM and fouling was compared with commercially available NF270 and NF90 membranes. The results revealed that HFNF (100–200 Da) and NF90 maintained considerably high rejection of Mn, Fe and NOM due to their dominant sieving effect. In contrary, the rejections of the above-mentioned components were decreased in the presence of high hardness for the looser HFNF (200–300 Da) and NF270 membranes. No membrane fouling was detected and the permeate flux was stable when the hard GW was filtered with the HFNF membranes, regardless of their molecular weight cut-off and transmembrane pressure, while the permeability of the NF270 and NF90 membranes steadily decline during the filtration. Integrating a calcite contactor, as a post filtration step, to the HFNF process yielded further Mn, Fe and NOM removals from the HFNF permeate and adjustment of its hardness level. The best performance was achieved when a blend of Calcite–CorosexTM (90/10wt.%) was used as a post-treatment to the tight HFNF (100–200 Da).

## 1. Introduction

Groundwater (GW) comprises a significant amount of all unfrozen fresh water and provides approximately half of the world’s drinking water supply. In North America, GW is the main drinking water source for nearly 44% of the United States population and 27% of Canadians, mostly living in remote and small communities. High levels of dissolved manganese (Mn) and iron (Fe) often naturally co-exist in GWs as a result of weathering and leaching of metal-bearing minerals and rocks [1]. Although, traditionally, aesthetic concerns (such undesirable color taste and odor, high turbidity and staining of laundry and plumbing fixture [1,2]) were the primary motivation for the Mn and Fe removal from the potable water sources, the findings of recent epidemiological studies addressed chronic neurological effects of elevated Mn level on school-age children [3,4,5]. These concerns prompted Health Canada to propose new maximum acceptable aesthetic (20μg Mn/L) and health (120μg Mn/L) levels for Mn in drinking water [6]. Likewise, the United States Environmental Protection Agency added Mn to the fourth Candidate Contaminant Candidate List [7].

**Figure 1:**
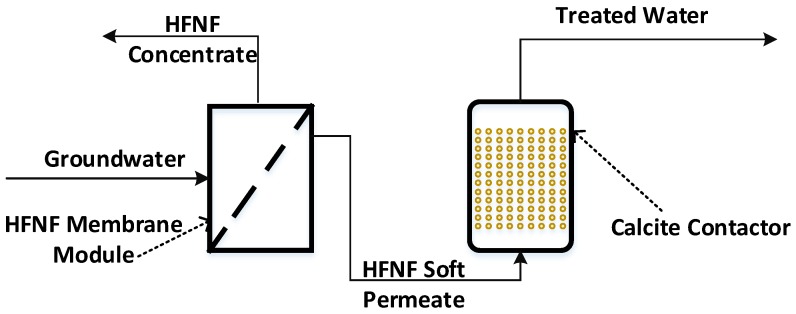
Schematic drawing of the investigated point-of-entry hybrid HFNF–calcite contactor process for the treatment of domestic GW supplies.

Common treatment methods for Mn and/or Fe removal from domestic GW sources are catalytic filtration and cationic ion exchange resins (in conjunction with a point-of-use reverse osmosis (RO) unit placed at the kitchen tap). However, complicated operation and maintenance along with the risk of Mn leaching in the former treatment process and removal of beneficial nutrients in the finished water as well as a high amount of salt consumption and production a spent brine in the latter approach are the main drawbacks of these options [8,9,10]. To tackle these concerns, we recently assessed the performance of hollow fiber nanofiltration (HFNF) membranes for the removal of dissolved Mn, Fe and natural organic matter (NOM) from GW suplies [11]. Our investigation demonstrated that in the absence of hardness above 90% of Mn, Fe and NOM were retained by the examined HFNF membranes; whereas increasing the hardness level of the GW lowered the removal of the Mn and Fe ions [11]. It was found that the presence of hardness in the form of Ca and/or Mg salts prompted the binding of these ions (i.e., Ca${}^{2+}$ and Mg${}^{2+}$) to the fixed charged groups on the surface of the HFNF membranes which weakened their rejection by charge exclusion. Evidently, the tested HFNF membranes were not tight enough to sieve the passage of the Mn and Fe ions. As opposed to Mn and Fe, which would be ideally fully removed from the source waters, it is not desirable to fully remove hardness in order to (1) avoid the production of excessively corrosive waters and (2) maintain a minimal Mg and Ca content in the finished water for the health purpose. This investigation was conducted to diminish the adverse impact of the GW hardness and complement the performance of the HFNF for the removal of Mn and Fe. In this way, we put forth the hypothesis that implementation of a point-of entry hybrid membrane system can lead to an effective and robust removal of dissolved Mn, Fe, hardness, NOM and pathogens from the GWs. In this proposed treatment system, a membrane module containing tighter HFNFs (with improved sieving properties) is coupled with a post-filtration step such as calcite (CaCO${}_{3}$) contactor (Figure 1). The use of the HFNF membranes is preferred over the classical spiral-wound nanofiltration (NF) membranes due to the chlorine resistance and backwashing features of these membranes. The calcite contactor, on the other hand, may play a dual function of (1) adjusting the hardness level of the HFNF permeate (as the membranes are non-selective and the permeate can be considerably soft and corrosive depending on the influent water characteristics) and (2) absorbing residual traces of Mn and Fe from the HFNF permeate (which were not completely retained during the NF step). If needed, magnesium oxide (MgO) can also be blended with calcite in order to add Mg and raise the pH of the finished water.

Despite the wealth of information on the effectiveness of implementing a calcite contactor to remineralize desalted RO permeates [12,13] and its ability to adsorb divalent metallic cations on its surface [14,15,16], to the best of our knowledge, removal of Mn and Fe from a soft water while simultaneously adjusting its hardness level (without dosing CO${}_{2}$) by means of a calcite contactor is not addressed in the scientific literature. In this perspective, the aim of this research is to design a simple and compact, yet robust hybrid HFNF–calcite contactor process for domestic treatment of GWs (containing a high level of Mn, Fe and NOM). The treated water should meet the recent Health Canada regulation on Mn level (target limit of 20μg Mn/L) with a hardness level not lower than 40 mg CaCO${}_{3}$/L and a pH above 8.0. To accomplish this goal, two specific objectives were defined: (i) evaluating the performance of tight HFNF membranes (under the applied transmembrane pressures (TMP) of 5–8 bars) on Mn and Fe removal from a GW containing NOM and a high level of hardness and (ii) determining the role of media configuration (pure CaCO${}_{3}$ vs. a blend of CaCO${}_{3}$ and MgO) and NF permeate composition on the efficiency of the calcite contactor to achieve simultaneous removal of Mn and Fe traces and hardness adjustment of the soft NF permeate.

## 2. Experimental

### 2.1. Materials

Four types of NF membranes were tested in this study including commercially available thin-film composite (TFC) polyamide membranes, NF270, and fully aromatic TFC polyamide membrane, NF90, from Dow Filmtec along with two outside-in TFC sulfonated polyethersulfone (SPES) HFNF membranes modules (provided by Toyobo, Osaka, Japan). Compared to the TFC polyamide membranes, the HFNF membranes were chlorine-resistant and backwashable and offer approximately four-fold higher packing density than spiral wound NFs. The main properties of the membranes are listed in Table 1.

Natural GW was collected from Sainte Marthe sur le Lac municipal wells (Quebec, Canada). To remove suspended solids, upon collection, the water was vacuum filtrated using a 0.45 μm hydrophilic polyethersulfone (PES) filter paper (Supor${}^{®}$ PES Membrane Disc Filters, Pall, New York, NY, USA). The specifications of the GW are summarized in Table 2. Prior to the filtration assays, Mn and Fe concentrations of the natural GW were artificially elevated to 1 mg/L by adding a pre-determined amount of MnSO${}_{4}$ and FeSO${}_{4}$ stock solutions to the deoxygenated GW in the feed tank.

Commercially available calcite (CaCO${}_{3}$) media from Imerys Marble Inc. (Sahuarita, AZ, USA) and Corosex${}^{\mathrm{TM}}$ (MgO) media from Clack Corporation, Windsor, Wisconsin, USA were used. The main characteristics of the media are given in Table 3. Note that both calcite and Corosex${}^{\mathrm{TM}}$ can increase the hardness level by adding Ca and Mg ions to the water, respectively [18].

### 2.2. Experimental Design

Two consecutive experimental phases were designed. During the first phase, the performance of the HFNF membranes and commercially available TFC polyamide NF270 and NF90 membranes were compared in terms of Mn, Fe and NOM retention and fouling at the TMP range of 5–8 bars. Based on the findings of the first phase, the focus of the second phase was to determine the impacts of the feed (i.e., NF soft permeate) composition and media characteristics on the removal of Mn, Fe and NOM traces and hardness adjustment of the potentially NF soft permeate. In this regard, two common remineralization media (i.e., calcite and Corosex${}^{\mathrm{TM}}$) were used. As the first series of the experiments, the column was filled with calcite media, while for the second set of the assays a mixture of calcite and Corosex${}^{\mathrm{TM}}$ media was tested in order to investigate the impact of the media specification on the quality of the finished water.

### 2.3. Protocols

#### 2.3.1. Nanofiltration

A lab-scale cross flow membrane filtration setup was employed in this investigation. The details of the filtration apparatus and protocol were explained in our previous study [11]. In brief, the filtration tests were undertaken at a constant pressure using mini HFNF modules and CF042 cells (Sterlitech, Kent, WA, USA) fitted with NF270 or NF90. Throughout the filtration experiments, the cross flow velocity and operating temperature were kept constant at 0.12±0.01 m/s and 10±1 ${}^{\circ }$C, respectively. The water temperature was selected based on the average GW temperature in southern Quebec, Canada. To maintain the feed specifications constant, the permeate and concentrate lines were sent back to the feed tank. Samples were collected every hour from the feed, concentrate and permeate sampling valves to monitor Mn, Fe, NOM and hardness levels and the temperature, pressure and flow rate of the feed, concentrate and permeate lines were recorded automatically every 20 s. Prior to and after each test, the pure water flux was measured and if needed, a four-step consecutive chemical cleaning (i.e., NaOH, ultrapure water, citric acid and ultrapure water) was carried out to wash/remove any foulants that might remain in the system and restore the membranes specifications for the next filtration test. Details on post chemical cleaning procedure can be found in [11]. Each filtration experiment was run for 6 h using the natural GW with three replicates.

#### 2.3.2. Post-Filtration via a Calcite Contactor

**Figure 2:**
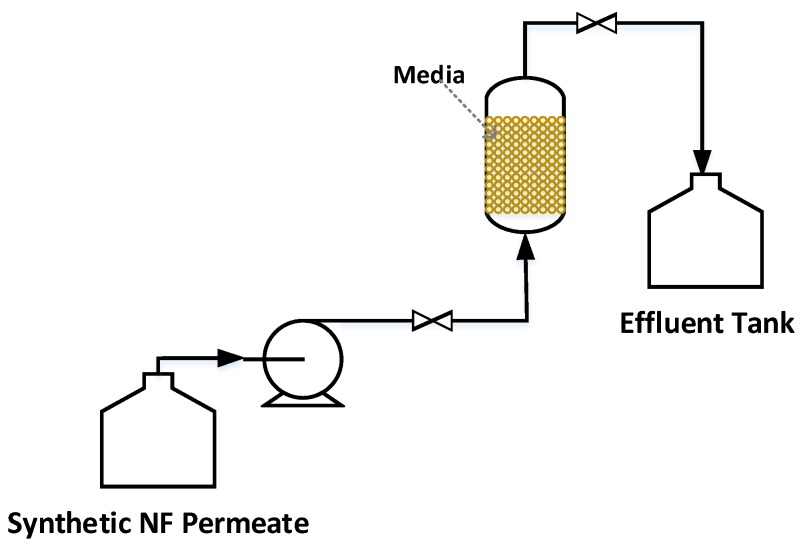
Schematic illustration of the calcite contactor set-up.

Simultaneous Mn and Fe removal and hardness adjustment of the synthetic NF soft permeate were done with a lab-scale up-flow column (media height of 20 cm and an internal diameter of 1.5 cm). To mimic the NF permeate specifications, synthetic solutions were prepared by adding appropriate amounts of the analytical grade MnSO${}_{4}$, FeSO${}_{4}$, CaSO${}_{4}$ and MgSO${}_{4}$ salts (purchased from Fisher Scientific, Fair Lawn, NJ, USA) and Suwannee River NOM (supplied by the International Humic Substances Society, St. Paul, MN, USA) to deoxygenated ultrapure water at pH =6.1±0.4. Based on the information found in the literature, the empty bed contact time was set at 15 min [16]. The feed water was pumped to the column and the effluent was collected from the top of the column (Figure 2). Each experiment was run for 3 h and samples were taken every 30 min via influent and effluent sampling valves. The pH, temperature and oxygen level of the feed tank were monitored continuously. All the assays were done three times at a constant temperature of 10±1 ${}^{\circ }$C.

### 2.4. Analytical Methods

Hardness was measured based on the standard titration method (i.e., Hardness (2340)/EDTA Titrimetric Method). Total and dissolved Mn, Fe, Ca and Mg concentrations of the feed, concentrate and permeate samples were determined by ICP-AES (Thermo Fisher, ICAP 6000). Details of the ICP sample preparation and the analytical procedure were elaborated in our previous work [11]. The NOM content of the samples was quantified as dissolved organic carbon (DOC) concentration and measured with a total organic carbon (TOC) analyzer (Model: Sievers M 5310 C, GE Instruments, Boulder, CO, USA).

### 2.5. Statistical Analyses

The experimental data are reported as means ± standard deviation and subjected to one-way and multiple-way statistical analyses with the common level of significance set at p=0.05.

## 3. Results and Discussion

### 3.1. Nanofiltration

#### 3.1.1. Membrane Permeability

**Figure 3:**
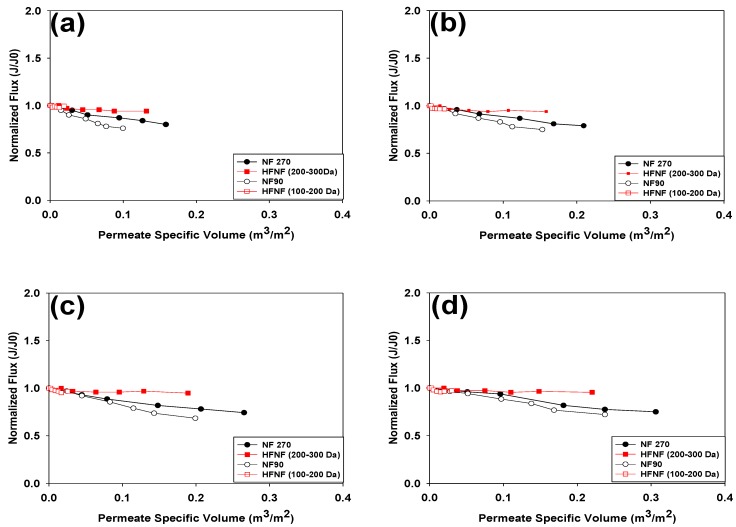
Permeate flux profiles of the tested NF membranes at (**a**) TMP = 5 bars, (**b**) TMP = 6 bars, (**c**) TMP = 7 bars and (**d**) TMP = 8 bars (filtration of the natural GW containing 1 mg/L of Mn and Fe, 0.33 mg C/L NOM and hardness level of 250 mg CaCO${}_{3}$/L at T = 10 ${}^{\circ }$C).

Figure 3 depicts the normalized permeate flux as a function of the specific permeate volume for the TMP range of 5–8 bars. Increasing the driving force by exerting a higher TMP led to a higher permeate flux which translates into high productivity (i.e., the specific permeate volume). In addition, one can note that filtration of the hard GW with the flat sheet membranes (i.e., NF 270 and NF90) and loose HFNF (200–300 Da) resulted in higher permeate specific volumes; nevertheless, the permeate flux of the NF270 and NF90 membranes declined significantly throughout the filtration experiments, regardless of the applied TMP (*p*= 0.03). The decrease in the normalized permeate flux was steeper when the hard GW was filtered with the NF90 membranes, for all the applied TMPs. On the other hand, both of the tested HFNF membranes exhibited a stable normalized permeate flux during the hard GW filtration tests. The lower normalized permeate flux of the HFNF (100–200 Da) can be linked to its lower MWCO. According to the information given in Table 1, the pure water flux of the HFNF (100–200 Da) was 7.8, 12 and 25 times lower compared to the HFNF (200–300 Da), NF90 and NF270 membranes, respectively.

The descending trend of the normalized permeate flux of the NF270 and NF90 membranes indicates that the membrane fouling occurred during the filtration of the hard GW. A number of parameters can contribute to the fouling of a membrane such as hydrodynamic conditions, feed composition and membrane properties [19]. Previous studies demonstrated that morphology of the membrane surface (in other words membrane roughness) may initiate the fouling phenomenon [20,21,22,23]. These studies indicated that the initial membrane fouling mainly took place in the valleys of the rough membranes. By a simple comparison between the surface roughness of the HFNF membranes (reported in our recent work [11]) and the surface roughness of the NF270 and NF90 (documented by [24]), one can conclude that the HFNF membranes presented a smoother surface which enhances the antifouling properties of these membranes. Consequently, a stable normalized permeate flux was observed when the hard GW was filtered via the HFNF membranes. Most probably, co-existence of Ca and NOM in the GW caused the membrane fouling as a number of researchers illustrated that the presence of Ca and NOM in the feed can lead to the formation of Ca-NOM complexes on the surface of the membrane and, subsequently, membrane fouling and reduction of the membrane permeability [25,26,27]. In summary, from the sole perspective of the membrane fouling and productivity, the HFNF (200–300 Da) membranes provided the most stable and superior performance amongst the four tested membranes.

#### 3.1.2. Mn, Fe, NOM and Hardness Removal

**Figure 4:**
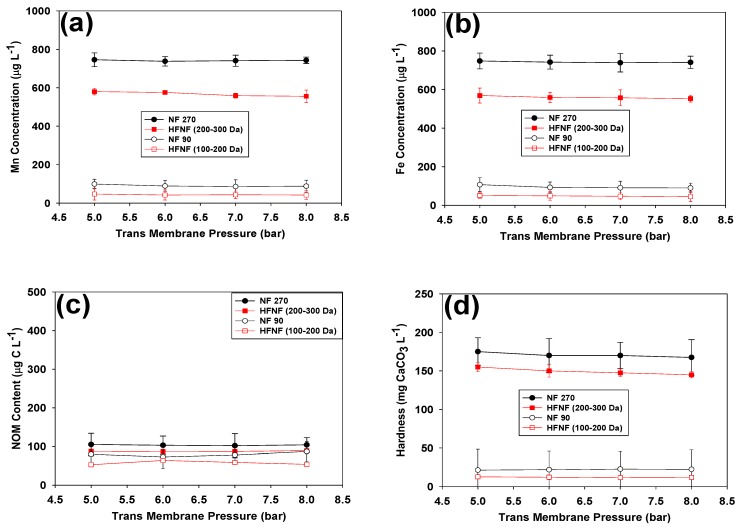
Mn, Fe, NOM and hardness levels of the permeate line of the tested NF membranes during the filtration process at (**a**) TMP = 5 bar, (**b**) TMP = 6 bar, (**c**) TMP = 7 bar and (**d**) TMP = 8 bar (filtration of the natural GW containing 1 mg/L of Mn and Fe, 0.33 mg C/L NOM and hardness level of 250 mg CaCO${}_{3}$/L at T = 10 ${}^{\circ }$C).

The performance of the tested membranes was further assessed by quantifying the removal of dissolved Mn, Fe, NOM and hardness during the filtration of the hard GW at TMP ranging from 5 to 8 bars (Figure 4). It is worth mentioning that, for all the tested conditions, the Mn and Fe concentrations of the permeate lines were stable throughout the filtration experiments. Contrarily, NOM and Ca levels of the feed solution slightly decreased (≤10%) during the filtration of the GW when the NF270 and NF90 membranes were tested (data not presented). As explained earlier, reductions of the NOM and Ca levels can be attributed to the formation of Ca-NOM complexes on the surface of these membranes and, accordingly, a decline in the permeate flux (Figure 3) [25,26,27]. For ease of comparison, the average values of Mn, Fe, NOM and hardness of the permeate lines were plotted in Figure 4. The error bars illustrate the standard deviation of the collected data.

When the tight NF membranes (i.e., NF90 and HFNF (100–200 Da)) were used, above 90% of Mn, Fe, NOM and hardness were removed from the GW. On the contrary, the membranes with higher MWCOs (i.e., NF270 and HFNF (200–300 Da)) only retained around 42% of Mn and Fe and up to 90% of NOM, independently of the applied TMP. The highest and lowest removal efficiencies were achieved with the HFNF (100–200 Da) and NF270 membranes, respectively. With an initial Mn concentration of 1 mg Mn/L in the feed, the Mn content of the permeate of the tight NF membranes (NF90 and HFNF(100–200 Da)) was still above our targeted concentration of 20μg/L.

As anticipated, the NF membranes were not selective and the implementation of the tight membranes not only yielded a high removal of dissolved Mn, Fe and NOM but also caused a significant depletion in the hardness level of the filtered water (Figure 4d), whereas an acceptable hardness level of the filtered water was achieved when the loose NF membranes (i.e., NF270 and HFNF (200–300 Da)) were employed at the cost of a lower Mn/Fe rejection.

In NF processes, the membranes retain the components mainly based on the size and/or charge exclusion(s) [28,29,30]. Previous studies showed that the presence of Ca and Mg in the feed can lower the charge density of the surface of the NF270 and NF90 membranes due to the binding propensity of these ions with the carboxylic fixed charged group on the surface of these membranes [17,31]. We, recently, demonstrated that the interaction between the Ca and Mg ions and fixed charged sulfonic groups on the surface of the HFNF membranes impairs their charge exclusion mechanism [11]. Accordingly, it can be deduced that, during the filtration of the hard GW, the size exclusion played the dominant retention role and, subsequently, significant removal of Mn, Fe, NOM and hardness was achieved when membranes with tighter pores (i.e., NF90 and HFNF (100–200 Da)) were applied.

Another interesting point that is worth mentioning here is the contribution of the type of the NF membranes ligand groups on their overall performance. Zhao and co-workers reported that Ca ions have a higher binding energy and stronger affinity to attach to the carboxylic groups (ligand groups of the NF270 and NF90 membranes) than sulfonic groups (ligand groups of the HFNF membranes) [32]. Thus, we can presume that the bound Ca ions to the carboxylic groups facilitated the formation of the Ca-NOM complexes on the surface of the NF270 and NF90 membranes and adversely impacted the productivity of these membranes in terms of fouling and removal efficiency.

### 3.2. Post-Filtration via Calcite Contactor

**Figure 5:**
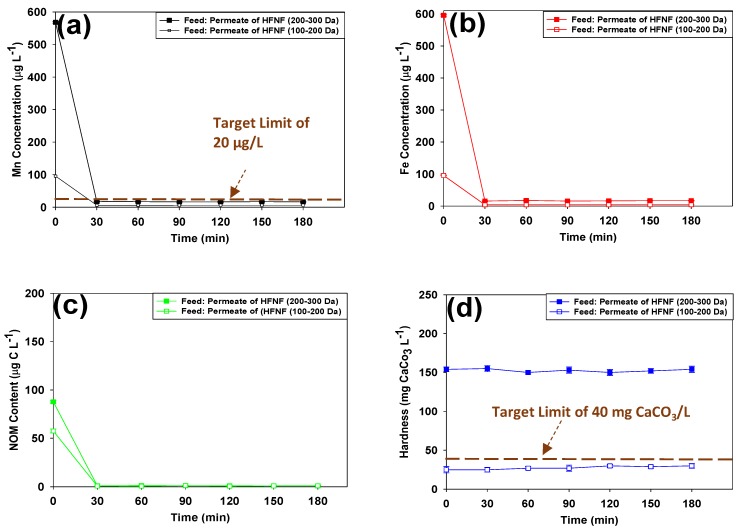
Levels of (**a**) Mn, (**b**) Fe, (**c**) NOM and (**d**) hardness at the effluent line of the calcite contactor during the post-filtration process at T = 10 ${}^{\circ }$C (Data at time = 0 provide the initial concentration of the components at the influent of the calcite contactor: The synthetic HFNF (200–300 Da) permeate composed of 0.568 mg Mn/L, 0.595 mg Fe/L, 0.087 mg C/L (expressed as NOM) and hardness level of 154 mg CaCO${}_{3}$/L, whereas the HFNF (100–200) permeate composed of 0.096 mg Mn/L, 0.095 mg Fe/L, 0.058 mg C/L (expressed as NOM) and hardness level of 25 mg CaCO${}_{3}$/L).

Based on the findings of the first experimental phase, it is evident that the HFNF membranes presented a superior performance than the commercially available NF270 and NF90 membranes in terms of fouling and removal efficiency. Therefore, synthetic feed solutions with characteristics similar to the HFNF (200–300 Da) and HFNF (100–200 Da) permeates were prepared to feed the lab-scale calcite contactor. Representative data in Figure 5 demonstrate the levels of Mn, Fe, NOM and hardness at the effluent of the calcite contactor during the post-filtration of the HFNF permeates. It is apparent that, under the applied operational conditions, the media efficiently removed Mn, Fe and NOM traces, regardless of their initial concentration in the feed solution (*p* = 0.03). On the other hand, the hardness of the soft HFNF (100–200 Da) was increased slightly; while no significant change was detected in the hardness of the HFNF (200–300 Da) permeate after its passage through the calcite contactor at an empty bed contact time of 15 min (*p* = 0.07).

Removal of Mn and Fe by the calcite media can be attributed to the displacement of the Ca ions by Mn and/or Fe ions as:

(1) $$\;CaCO_{3(S)}+Me_{\left(eq\right)}^{2+}⟹MeCO_{3}+Ca_{\left(eq\right)}^{2+}$$

In this equation, Me${}^{2+}$ represents the metal ion present in the solution. Zachara et al. stated that metals with ionic radii smaller than Ca are more prone to adsorb onto the media. It is worth mentioning that the attached Me${}^{2+}$ to the calcite media exhibits a very slow desorption rate which favors the application of the calcite contactor for the Mn removal from the NF permeate [33]. Furthermore, it is assumed that the formation of the Ca-NOM complexes led to the removal of NOM traces (≤100 mg C/L) during the post-filtration process.

The evolution of the hardness profiles of the effluent streams can be explained by the solubility of calcium carbonate in clean waters. Stumm and Morgen [34] reported that the solubility of calcium carbonate is around 47 mg CaCO${}_{3}$/L at atmospheric CO${}_{2}$. Hence, we can postulate that the empty bed contact time of 15 min was not long enough to elevate the hardness level of the HFNF (100–200 Da) permeate even up to the maximum level of the calcite solubility. As for the HFNF (200–300 Da) permeate, its initial hardness level was already higher than the maximum solubility of calcite and with such a high initial hardness level (i.e., 150 mg CaCO${}_{3}$/L), no further hardness adjustment would be required for the HFNF (200–300 Da) permeate. Thus, in this case, the role of the calcite contactor would be solely capturing the Mn, Fe and NOM traces. Moreover, feeding the calcite contactor with supersaturated hardness is not desirable as media clogging can be expected during long-term operation due to calcite precipitation from the feed. Therefore, we conclude that the use of the tighter HFNF (100–200 Da) membrane would be preferable to achieve the feed water calcium concentration below the calcite solubility.

The calcite contactor, on the other hand, was not effective to increase the hardness of the HFNF (100–200 Da) permeate to our target level of 40 mg CaCO${}_{3}$/L. The efficacy of the calcite contactor to adjust the hardness of a soft water can be enhanced by: (1) increasing the empty bed contact time, (2) adding CO${}_{2}$ to the feed solution in order to increase calcite solubility [34] or (3) blending the calcite media with another media such as Corosex${}^{\mathrm{TM}}$ (i.e., MgO) which can increase the water hardness. Prolonging the contact time of the feed water with the media is not an economically attractive option as the investment cost of the process would increase. Improved performance of the calcite beds by CO${}_{2}$ injection is mentioned in the literature [12,13]. However, for purpose of the domestic GW treatment, CO${}_{2}$ injection complicates the operation and maintenance of the process and would also add an additional cost. Thus, in the course of this study, the effectiveness of a blend of the calcite-Corosex${}^{\mathrm{TM}}$ in simultaneous removal of Mn, Fe, NOM and hardness adjustment of the soft NF permeate was investigated.

**Figure 6:**
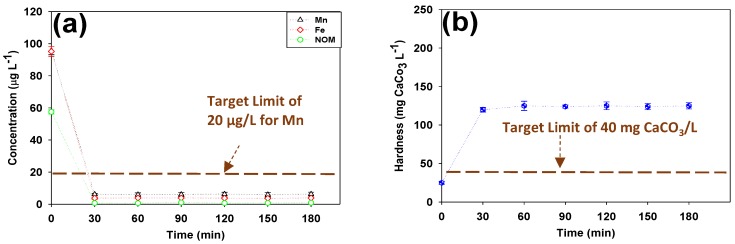
Levels of (**a**) Mn, Fe, NOM and (**b**) hardness of at the effluent line of the calcite-Corosex${}^{\mathrm{TM}}$ contactor during the post-filtration process at T = 10 ${}^{\circ }$C (Data at time = 0 provide the initial concentration of the components at the influent of the calcite contactor: The synthetic HFNF (100–200 Da) permeate composed of 0.096 mg Mn/L, 0.095 mg Fe/L, 0.058 mg C/L (expressed as NOM) and hardness level of 25 mg CaCO${}_{3}$/L).

The soft HFNF (100–200 Da) permeate was passed through a mixed bed of calcite-Corosex^TM^ (90/10wt.%) and the Mn, Fe and NOM contents of the effluent were plotted during the post-filtration experiment (Figure 6a). As can be seen, blending the calcite and Corosex${}^{\mathrm{TM}}$ media had no adverse impact on the Mn and Fe removals and levels of these elements in the filtered water were very low (≤10μg/L) (*p* = 0.02). Moreover, throughout the post-filtration of the soft HFNF (100–200 Da) permeate, the hardness of the finished water reached a stable and acceptable level of 122 ± 2 mg CaCO${}_{3}$/L (Figure 6b). As expected, Corosex${}^{\mathrm{TM}}$ media elevated the hardness of the soft NF-permeate while the Mn, Fe and NOM traces were captured by the calcite media (based on the mechanism explained earlier). It should be noted that the blended media increased the pH of the filtered water to 9.4 ± 0.2 which is well below the maximum accepted limit proposed by Health Canada (i.e., 10.5).

### 3.3. Implications

According to the experimental findings, it is evident that the MWCO of the membrane has a pronounced influence on the retention of Mn and Fe ions when a hard GW is filtered. As expected, the tighter the membrane the higher the retention. Nonetheless, in the case of a high initial concentration of Mn and Fe in the GW sources (investigated in this study) the desired target limit of these components may not be achieved by a standalone HFNF process and employing a calcite-Corosex${}^{\mathrm{TM}}$ contactor, as a post-filtration step, was inevitable to diminish the Mn and Fe concentrations and increase the hardness/pH of the treated waters. The role of the calcite contactor in the Mn and Fe removal becomes more significant when the hard GW was filtered with the looser NF membranes.

Depending on the feed water characteristics, the treatment of the domestic GWs by means of the hybrid HFNF–calcite contactor can be undertaken in two configurations: (1) loose HFNF membranes coupled with a calcite contactor or (2) tight HFNF membranes in conjunction with a calcite-Corosex${}^{\mathrm{TM}}$ (90/10wt.%) contactor. Despite the fact that implementation of the former configuration would lead to a higher permeate flux, its application would be restricted to a feed water with a low to moderate hardness content of approx. 60 mg CaCO${}_{3}$/L (assuming that 40% rejection takes place during the HFNF step and the finished water reaches a maximum hardness of 47 mg CaCO${}_{3}$/L). Whereas, for waters with higher hardness, we propose the latter configuration since, in a long term operating of the calcite contactor, precipitation of the excess calcium carbonate (due to the high initial concentration in the inlet of the contactor) might cause operational problems. Thereby, further investigations on the improvement of the permeability of the tight HFNF membranes while maintaining its superior retention and antifouling properties are recommended. It is worth highlighting that, it is anticipated that HFNF (100–200 Da) membranes would provide an effective disinfection barrier against viruses (which is also a common GW treatment objective); nonetheless, this consideration was out of the scope of the current investigation.

From a practical standpoint, the implementation of an integrated HFNF (100–200 Da)–calcite contactor would most likely require the installation of a booster pump prior to the membrane as the available pressure from a well pump (in the range of 3–5 bars) is expected to be very low for a directly feeding of the membrane module. To provide a sufficient flow for a single residential dwelling (i.e., Q=1200 L/d, a small HFNF membrane module (H =1.0 m, ID=3.4 cm) with 10 m${}^{2}$ of membrane area (14469 fibers of 220 μm OD and packing density of 60%) is required. In addition, a calcite contactor with 15 min empty bed contact time would require a reactor of approx. 1.25 m high (ID=0.15 m). Finally, a hydropneumatic reservoir should be used in order to supply the peak water demand. Overall, the design of the treatment train would be compact. Future studies should focus on the optimization of the HFNF membrane operational conditions (i.e., cross flow conditions and recovery) in order to improve the overall performance of this treatment process.

## 4. Conclusions

This study sought to evaluate the performance of a hybrid HFNF–calcite contactor process (as an alternative treatment process) for simultaneous removal of Mn, Fe, NOM and hardness from domestic hard GW with regard to a high removal efficiency and minimum membrane fouling. Based on the obtained results, the following conclusions can be drawn:

- The HFNF (200–300 Da) and HFNF (100–200 Da) membranes offered a higher productivity than the NF270 and NF90 membranes which were tested as the reference cases.
- The HFNF (200–300 Da) and HFNF (100–200 Da) membranes exhibited a stable permeate flux during the filtration of the hard GW whereas the permeate flux of the NF270 and NF90 membranes considerably decreased due to the formation of Ca-NOM complexes on the surface of these membranes, regardless of the employed TMP.
- Size exclusion played the main role in retaining the dissolved Mn and Fe during the filtration of the hard GW: the presence of hardness negatively impacted charge exclusion by binding of the Ca and Mg ions to the charged groups on their surface. Thus, higher Mn and Fe removals were achieved when NF90 and HFNF (100–200 Da) were used.
- The surface morphology and type of the ligand groups on the surface of the NF membranes can initiate the membrane fouling phenomenon. The smoother surface of the HFNF membranes and presence of the sulfonic groups on their surface boosted their antifouling properties.
- When the tight NF membranes (i.e., NF90 and HFNF (100–200 Da)) were examined, above 90% of Mn, Fe were removed from the hard GW, while the Mn and Fe removal efficiency of the loose NF270 and HFNF (200–300 Da) was not greater than 42%. The HFNF (100–200 Da) and NF270 membranes exhibited the highest and lowest Mn and Fe retention, respectively.
- All the tested membranes could efficiently retain NOM as a result of charge and size exclusions.
- In the case of high initial concentrations of Mn and Fe in a hard GW, treatment with a standalone tight HFNF membranes might not lead to the desired target limit for these ions (especially the targeted Mn of 20μg/L) and a post-filtration step by means of a calcite-Corosex^TM^ (90/10 wt.%) contactor is required to further decrease the Mn and Fe levels in the finished water and adjust its hardness level.

## Figures and Tables

**Table 1:** Properties of the tested NF membranes (information provided by the supplier and/or extracted from the literature).

Membrane	Charged Groups	Membrane Effective Area (m${}^{2}$)	Pure Water Flux (LMH/bar)	MWCO (Da) *
NF270	COO${}^{-}$	0.0042	16.10 [17]	226 [17]
NF90	COO${}^{-}$	0.0042	7.69 [17]	118 [17]
HFNF	SO${}_{3}^{-}$	0.1750	5.00	200–300
	SO${}_{3}^{-}$	0.1710	0.64	100–200

* The reported Molecular weight cut-off (MWCO) is an estimation as it depends on the test conditions as well as charge, polarity, size and shape of the compound.

**Table 2:** Characteristics of the GW collected from Sainte Marthe sur le Lac municipal wells (the measurement were done after the vacuum filtration step).

Mn	Fe	Ca	Mg	Na	NOM *	Dissolved O${}_{2}$	pH	Hardness	Alkalinity
(mg/L)	(mg/L)	(mg/L)	(mg/L)	(mg/L)	(mg C/L)	(mg/L)		mg CaCO${}_{3}$/L	mg CaCO${}_{3}$/L
0.36±0.02	0.05±0.03	64.1±4.7	22.0±1.3	67.3±2.1	0.33±0.03	2.9±1.0	7.0±0.2	250.0±5.0	170.0±2.0

* Measured as dissolved organic carbon.

**Table 3:** Media Characteristics provided by the suppliers.

Media	D${}_{50}^{*}$ (mm)	Purity (%)	Specific Gravity (g/cm${}^{3}$)	Bulk Density (kg/cm${}^{3}$)	Surface Area (m${}^{2}$/g)
Calcite	0.4	99.7	2.68	1500	0.37
Corosex${}^{\mathrm{TM}}$	1.6	98.0	3.60	1200	0.39

D${}_{50}^{*}$: Median media diameter, measured in the laboratory by sieving the dry media.
